# Emerging Role of PPAR-**β**/**δ** in Inflammatory Process Associated to Experimental Periodontitis

**DOI:** 10.1155/2011/787159

**Published:** 2011-11-01

**Authors:** Rosanna Di Paola, Francesco Briguglio, Irene Paterniti, Emanuela Mazzon, Giacomo Oteri, David Militi, Giancarlo Cordasco, Salvatore Cuzzocrea

**Affiliations:** ^1^IRCCS Centro Neurolesi “Bonino-Pulejo”, 98125 Messina, Italy; ^2^Istituto Policattedra di Odontostomatologia Universita degli Studi di Messina, 98100 Messina, Italy; ^3^Department of Clinical and Experimental Medicine and Pharmacology, School of Medicine, University of Messina, Torre Biologica, Policlinico Universitario Via C. Valeria, Gazzi, 98100 Messina, Italy

## Abstract

The aim of the present study was to evaluate the contribution of peroxisome proliferator-activated receptor (PPAR-**β**/**δ**) in animal model of periodontitis. Male Sprague-Dawley rats were lightly anaesthetized with pentobarbitone (35 mg/kg). Sterile, 2-0 black braided silk thread was placed around the cervix of the lower left first molar and knotted medially. Animals received GW0742 (0.3 mg/kg, 10% DMSO, i.p. after the ligature placement and daily for eight days). At day 8, the gingivomucosal tissue encircling the mandibular first molar was removed. One the eighth day after placement of the ligature, we evaluated (1) NF-**κ**B expression, (2) cytokines expression, (3) iNOS expression, (5) the nitration of tyrosine, (6) apoptosis, and (8) the degree of gingivomucosal tissues injury. Administration of GW0742 significantly decreased all of the parameters of inflammation as described above. Taken together, these results demonstrate that GW0742 exerts an anti-inflammatory role during experimental periodontitis and is able to ameliorate the tissue damage.

## 1. Introduction

Periodontal disease is an inflammatory process involving progressive, episodic loss of the periodontal attachment apparatus, resulting ultimately in tooth loss in susceptible patients.

The initiation and progression of periodontal disease depend on the presence of pathogenic bacteria, host response, and risk factors. These risk factors encompass systemic influences (such as poorly controlled or uncontrolled diabetes mellitus), external influences (such as smoking), intrinsic factors, and local factors. They include oral hygiene, gender, race, socioeconomic status, age, systemic health status, use of medications, smoking, and alcohol and drug abuse. 

The inflammatory response in periodontal disease includes the activation of leucocytes, neutrophils, T lymphocytes, and plasma cells and the release of antibodies and chemical inflammatory mediators that include cytokines, chemokines, and C-reactive protein [1]. 

The initial increased presence of neutrophils at the site is followed by the release of cytokines by neutrophils and macrophages. Chemical mediators released include tumor necrosis factor alpha (TNF-*α*), interleukin-1 (IL-1), and prostaglandins [2]. The inflammatory process includes the stimulation of fibroblasts by IL-1 and the secretion of matrix metalloproteinases (MMPs, of which collagenase is the most prominent) by polymorphonuclear neutrophils. MMPs are responsible for increased collagen breakdown, and TNF-*α* is primarily responsible for increased osteoclast activity resulting in bone resorption [3]. T lymphocytes secrete receptor activator of nuclear factor kappa-B ligand (RANKL), which is involved in osteoclast activity and, therefore, bone resorption [4].

The family of transcription factors termed peroxisome proliferator-activated receptors (PPARs) has recently been the focus of much interest for their possible role in the regulation of inflammation and immune responses [5]. In particular, PPAR*α* and PPAR*γ* inhibit the activation of inflammatory gene expression and can negatively interfere with proinflammatory transcription factor signalling pathways in vascular and inflammatory cells. In contrast, the roles of PPAR*β*/*δ* regulating inflammation and immunity are only just emerging [6]. 

In general, PPARs must be activated by ligands to stimulate the expression of their target genes. These agonists can be synthetic molecules, such as drugs used to treat hypertriglyceridemia and insulin resistance, or natural physiological ligands, such as fatty acids and eicosanoids [7]. GW0742, can act as ligand of PPAR-*β*/*δ*. In particular, it has been reported that PPAR-*β*/*δ* ligands can inhibit the expression of various proinflammatory cytokines, such as TNF-*α* and IL-1*β*, vascular cell adhesion molecule-1, platelet-activating factor (PAF) receptor, and cyclooxygenase (COX)2 generation [8].

In this study, we wanted to investigate whether the modulation of the inflammatory process could limit the development of periodontitis analyzing the effects of GW0742 a synthetic high-affinity ligand for PPAR-*β*/*δ*. In particular, to gain a better insight into the mechanism(s) of action, we have studied the effect of the PPAR-*β*/*δ* agonist on the following endpoints of the inflammatory response: (1) histological damage, (2) bone loss (radiography), (3) cytokine expression (4) nitrotyrosine and inducible nitric oxide synthase (iNOS) expression, and (5) apoptosis.

## 2. Materials and Methods

### 2.1. Surgical Procedure

Male Sprague-Dawley rats (280–400 g) were lightly anaesthetized with surgical doses of sodium pentobarbitone (35 mg/kg). Sterile, 2-0 black braided silk thread was placed around the cervix of the lower left first molar and knotted medially as previously described [9]. After the rats had recovered from the anaesthetic, they were allowed to eat commercial laboratory food and drink tap water ad libitum. Animal care and protocol were in compliance with the Italian regulations on protection of animals used for experimental and other scientific purposes (D.M. 116192) as well as with the EEC regulations (O.J. of E.C. L 358/1 12/18/1986). The animals and the study protocol were approved by the Institutional Animal Care and User Committee of the University of Messina, Messina, Italy.

### 2.2. Experimental Groups

Rats were randomly allocated into the following groups.

- Ligature + vehicle group: rats were subjected to ligature-induced periodontitis, and animals received vehicle i.p. (10% DMSO 1 h after the ligature placement and daily treatment for eight days).
- Ligature + GW0742 group: rats were subjected to ligature-induced periodontitis, and animals received GW0742 (0.3 mg/kg, 10% DMSO, i.p. after the ligature placement and daily for eight days).

At 8 days after the ligature induction of periodontitis, the rats (*N* = 10 from each group for each parameter) were sacrificed in order to assess the effects of the compound on an acute lesion. The right side that is not subject to ligature was used as control. 

In a separate set of experiments was included a dose response in order to evaluate the different effects of treatment with GW0742. The following groups Were used for this purpose.

- Ligature + vehicle group: rats were subjected to ligature-induced periodontitis, and animals received vehicle i.p. (10% DMSO 1 h after the ligature placement and daily treatment for eight days).
- Ligature + GW0742 group: rats were subjected to ligature-induced periodontitis, and animals received GW0742 (0.1 mg/kg, 10% DMSO, i.p. after the ligature placement and daily for eight days).
- Ligature + GW0742 group: rats were subjected to ligature-induced periodontitis, and animals received GW0742 (0.03 mg/kg, 10% DMSO, i.p. after the ligature placement and daily for eight days).

### 2.3. Histological Examination

For histopathological examination, biopsies of gingiva and mucosa tissue from the buccal and lingual aspect of the teeth were taken 8 days after the ligature induction of periodontitis. The tissue slices were fixed in 10% neutral-buffered formaldehyde for 5 days, embedded in paraffin, and sectioned. The sections, orientated longitudinally from the teeth crowns, were stained with trichrome and haematoxylin-eosin stains. In the gingivomucosal sections stained with trichrome stain, the total number of infiltrating leukocytes (e.g., neutrophils and mononuclear cells) in cortical interstitial spaces from gingiva and mucosa tissues was assessed quantitatively by counting the number of infiltrating leukocytes in 20 high-power fields.

### 2.4. Radiography

A radiographic examination at the eighth day after ligature placement was determinate as previously described [9]. Mandibles were placed on a radiographic box at a distance of 90 cm from the X-ray source. Radiographic analysis of normal and ligated mandibles was performed by X-ray machine (Philips X12 Germany) with a 40 kW exposure for 0.01 sec.

### 2.5. Measurement of Vascular Permeability by the Evans Blue Extravasations

Vascular permeability was determinate as previously described [10]. Briefly, animals received the Evans blue (2.5% dissolved in physiological saline, at a dose of 50 mg/kg) via a femoral venous catheter. Extravasated Evans blue in the excised gingivomucosal tissue samples was extracted with 1 mL formamide for 48 h at room temperature for spectrophotometric determination at 620 nm and expressed as *μ*g/g gingivomucosal tissue [10].

### 2.6. Measurement of Alveolar Bone Loss

The distance from the cementoenamel junction of the first lower molars to the alveolar crest was measured with a modification of the method. Recordings were made along the median axis of the lingual surface of the mesial and mediolingulal roots of the lower first left and right molars as previously described. These measurements were performed by an independent investigator who was unaware of the treatment regimens. The alveolar bone loss induced by the ligature was expressed as a difference between the left and the right sides.

### 2.7. Myeloperoxidase Activity

Myeloperoxidase activity, an indicator of polymorphonuclear leukocyte (PMN) accumulation, was determined in gingivomucosal tissue, as previously described [11]. Myeloperoxidase activity was defined as the quantity of enzyme degrading 1 *μ*mol/min of peroxide at 37°C and was expressed in milliunits/g of wet tissue.

### 2.8. Immunohistochemical Localization of IL-1*β*, iNOS, Nitrotyrosine, Fas-L, Bax, and Bcl-2

At the end of the experiment, the tissues were fixed in 10% (w/v) PBS-buffered formaldehyde, and 8 *μ*m sections were prepared from paraffin-embedded tissues. After deparaffinization, endogenous peroxidase was quenched with 0.3% (v/v) hydrogen peroxide in 60% (v/v) methanol for 30 min. The sections were permeabilized with 0.1% (w/v) Triton X-100 in PBS for 20 min. Nonspecific adsorption was minimized by incubating the section in 2% (v/v) normal goat serum in PBS for 20 min. Endogenous biotin- or avidin- binding sites were blocked by sequential incubation for 15 min with biotin and avidin (DBA, Milan, Italy), respectively. Sections were incubated overnight with antinitrotyrosine rabbit polyclonal antibody (1 : 500 in PBS, v/v) or with anti-iNOS antibody (1 : 500 in PBS, v/v) or 5), with anti-IL-1*β* polyclonal antibody (Santa Cruz Biotechnology, 1 : 500 in PBS, v/v), with anti-Fas-L polyclonal antibody (Santa Cruz Biotechnology, 1 : 500 in PBS, v/v), with anti-Bax polyclonal antibody (Santa Cruz Biotechnology, 1 : 500 in PBS, v/v), or with anti-Bcl-2 polyclonal antibody (Santa Cruz Biotechnology, 1 : 500 in PBS, v/v). Sections were washed with PBS and incubated with secondary antibody. Specific labelling was detected with a biotin-conjugated goat anti-rabbit IgG and avidin-biotin peroxidase complex (DBA, Milan, Italy). In order to confirm that the immunoreactions for the nitrotyrosine were specific, some sections were also incubated with the primary antibody (antinitrotyrosine) in the presence of excess nitrotyrosine (10 mM) to verify the binding specificity. To verify the binding specificity for Bax, Bcl-2, iNOS, and IL-1*β*, some sections were also incubated with only the primary antibody (no secondary) or with only the secondary antibody (no primary). In these situations, no positive staining was found in the sections indicating that the immunoreaction was positive in all the experiments carried out. Immunocytochemistry photographs (*n* = 5 photos from each sample collected from all rats in each experimental group) were assessed by densitometric analysis by using Optilab Graftek Software on a Macintosh personal computer.

### 2.9. Western Blot Analysis for IkB-*α*, NF-*κ*B, iNOS, Bax, and Bcl-2

In brief, gingivomucosal tissues from each rat were suspended in extraction buffer A containing 0.2 mM phenylmethylsulfonyl fluoride (PMSF), 0.15 *μ*M pepstatin A, 20 *μ*M leupeptin, and 1 *μ*M sodium orthovanadate, homogenized at the highest setting for 2 min, and centrifuged at 1000 g for 10 min at 4°C. Supernatants represented the cytosolic fraction. The pellets, containing enriched nuclei, were resuspended in buffer B containing 1% Triton X-100, 150 mM NaCl, 10 mM Tris-HCl, pH 7.4, 1 mM EGTA, 1 mM EDTA, 0.2 mM PMSF, 20 M leupeptin, and 0.2 mM sodium orthovanadate. After centrifugation at 15,000 g for 30 min at 4°C, the supernatants containing the nuclear protein were stored at −80°C for further analysis. Protein concentration was determined with the by Bio,Rad Protein Assay (Bio-Rad, Milan, Italy). The levels of IkB-*α*, iNOS, Bax, and Bcl-2 were quantified in cytosolic fraction, while NF-*κ*B p65 levels were quantified in nuclear fractions. The membranes of nitrocellulose were blocked with 1x PBS, 5% (w/v) nonfat dried milk for 40 min at room temperature, and they were subsequently probed with specific antibodies IkB-*α* (1 : 1000; Santa Cruz Biotechnology, Inc.), anti-iNOS (1 : 1000 Signal Transduction), anti-Bax (Santa Cruz Biotechnology, 1 : 500), and anti-Bcl-2 (Santa Cruz Biotechnology, 1 : 500) or anti- NF-*κ*B p65 (1 : 1000; Santa Cruz Biotechnology) in 1x PBS, 5% (w/v) nonfat dried milk, and 0.1% Tween 20 at 4°C overnight. Membranes were incubated with peroxidase-conjugated bovine antimouse IgG secondary antibody or peroxidase-conjugated goat anti-rabbit IgG (1 : 2000; Jackson Immuno Research Laboratories Inc., West Grove, Pa, USA) for 1 h at room temperature. To ascertain that blots were loaded with equal amounts of protein lysates, they were also incubated in the presence of the antibody against *β*-actin (1 : 10,000 Sigma-Aldrich Corp.). The relative expression of the protein bands of I*κ*B-*α* (~37 kDa), iNOS (~130 kDa), NF-*κ*B p65 (65 kDa), Bax (~23 kDa), Bcl-2 (~29 kDa) was quantified by densitometric scanning of the X-ray films with GS-700 Imaging Densitometer (GS-700, Bio-Rad Laboratories, Milan, Italy) and a computer program (Molecular Analyst, IBM). 

### 2.10. Measurement of Cytokines

Gingivomucosal tissues were homogenized in PBS containing 2 mmol/L of phenyl-methyl sulfonyl fluoride (Sigma Chemical Co., Milan, Italy) and tissue levels of TNF-*α* and IL-1*β* were evaluated. The assay was carried out by using a colorimetric, commercial kit (Calbiochem-Novabiochem Corporation, USA) according to the manufacturer instructions. All cytokine determinations were performed in duplicate serial dilutions. Results are expressed as pg/100 g wet tissue.

### 2.11. Materials

The primary antibodies directed at Bax and Bcl-2 were obtained from Santa Cruz Biotechnology, Inc. (Santa Cruz, Calif, USA). The secondary antibody was obtained from Jackson ImmunoResearch Laboratories, Inc. (Jackson, Bar Harbor, Maine, USA). Unless otherwise stated, all compounds were obtained from Sigma-Aldrich Company Ltd (Me, Italy). All other chemicals were of the highest commercial grade available. All stock solutions were prepared in nonpyrogenic saline (0.9% NaCl; Baxter, Italy, UK). 

### 2.12. Statistical Evaluation

All values in figures and text are expressed as mean ± standard error (S.E.M.) of the mean of *n* observations. For the in vivo studies, *n* represents the number of animals studied. In the experiments involving histology or immunohistochemistry, the figures shown are representative of at least three experiments (histological or immunohistochemistry coloration) performed on different experimental days on the tissue sections collected from all the animals in each group. The results were analyzed by one-way ANOVA followed by a Bonferroni post-hoc test for multiple comparisons. A *P* value less than 0.05 was considered significant. And individual group means were then compared with Student's unpaired *t*-test. A *P* value of less than 0.05 was considered significant.

## 3. Results

### 3.1. Effect of GW0742 on Tissue Damage and Bone Resorption

**Figure 1:**
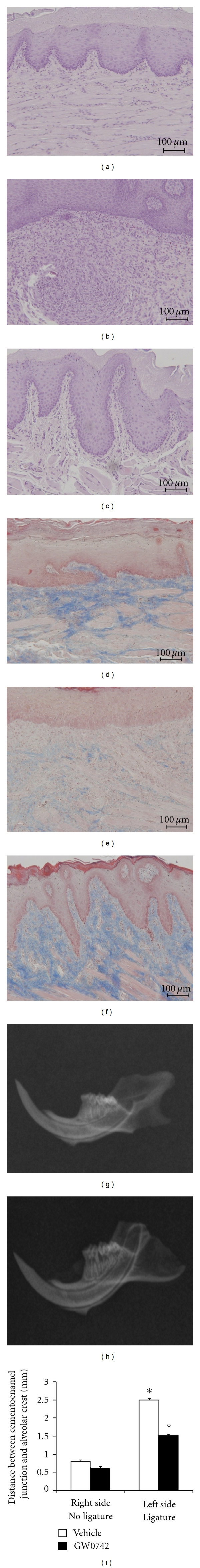
Effect of GW0742 on tissue damage and bone resorption. Inflammatory cells infiltration and edema were observed in gingivomucosal section from ligated rats (b) when compared with gingivomucosal tissue section taken from contralateral side (a). Significantly less edema and inflammatory cell infiltration was observed in gingivomucosal sections from ligature-treated rats which had been treated with GW0742 (c). Moreover, Masson's trichrome stain was negative in gingivomucosal tissue sections taken from the contralateral side of vehicle when compared with gingivomucosal tissues sections of ligature-operated rats (d, e, resp.). GW0742 treatment reduced the increase of collagen (f). The alveolar bone from ligated rats demonstrated alveolar bone resorption (g). GW0742 treatment suppressed alveolar bone resorption (h). A significant increase in the distance between cementoenamel injunction and alveolar crest at mediolingulal root of the first molar was observed in ligature-treated rats (i). GW0742 treatment significantly reduced the increase in the distance between cementoenamel injunction and alveolar crest (i). Figures are representative of at least 3 experiments performed on different experimental days. The tissue sections, orientated longitudinally from the teeth crown, were stained with trichrome stain. Data represent 20 counts obtained from the gingivomucosal tissue of each treatment group. *P < 0.01 versus nonligated. °*P* < 0.01 versus ligated.

When compared to gingivomucosal tissues sections taken from the contralateral side obtained from vehicle-treated rats (Figure 1(a)), histological examination of gingivomucosal tissues sections of ligature-operated rats showed oedema, tissue injury, as well as infiltration of the tissue with inflammatory cells (Figure 1(b)). GW0742 treatment reduced the degree of gingivomucosal tissues injury (Figure 1(c)). Moreover, Masson's trichrome stain, which is used to monitor the increase of collagen fiber, was negative in gingivomucosal tissue sections taken from the contralateral side from vehicle when compared with gingivomucosal tissue sections of ligature-operated rats (Figures 1(d) and 1(e), resp.). GW0742 treatment reduced the increase of collagen (Figure 1(f)).

A radiographic examination of the mandibles, at day 8 after ligature placement, revealed bone matrix resorption in the lower left first molar region after ligation (Figure 1(g)). There was no evidence of pathology in the right first molar (data not shown). GW0742 markedly reduced the degree of bone resorption in the lower left first molar region after ligation (Figure 1(h)). A significant alveolar bone loss between the lower first left molar and the right first molars induced by the left-side ligature was observed in vehicle-treated rats. GW0742 treatment resulted in a significant inhibition of alveolar bone loss after ligation (Figure 1(i)). Data represent the mean ± S.E.M. for 20 counts obtained from the gingivomucosal tissue of each treatment group *P < 0.01 versus nonligated. °*P* < 0.01 versus ligated. 

### 3.2. Effects of GW0742 Treatment on NF-*κ*B Activation in Periodontitis

**Figure 2:**
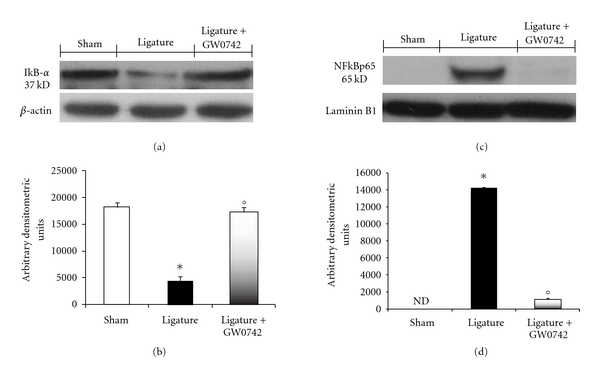
Effects of GW0742 treatment on NF-*κ*B activation in periodontitis. A basal level of I*κ*B-*α* was detected in the gingivomucosal tissue sections taken from the contralateral side (a, b). I*κ*B-*α* levels were substantially reduced (a, b) in the gingivomucosal tissues from ligature-operated rats. GW0742 treatment prevented I*κ*B-*α* degradation, (a, b). Periodontitis caused a significant increase in the NF-*κ*B p65 levels in the gingivomucosal tissues from operated rats (c, d). GW0742 treatment significantly prevented NF-*κ*B p65 expression (c, d). A representative blot of lysates obtained from 5 animals per group is shown and densitometry analysis of all animals is reported. The results in (b, d) are expressed as mean ± S.E.M. from *n* = 5/6 gingivomucosal tissues for each group. *P < 0.01 versus nonligated group. °*P* < 0.01 versus ligated group.

We evaluated I*κ*B-*α* degradation by Western Blot analysis to investigate the cellular mechanisms by which treatment with GW0742 may attenuate the development of periodontitis. A basal level of I*κ*B-*α* was detected in the gingivomucosal tissues from the contralateral side obtained from vehicle-treated rats, whereas in the gingivomucosal tissues from ligature-operated rats I*κ*B-*α* levels were substantially reduced (Figures 2(a) and 2(b)). GW0742 treatment prevented the ligature-induced I*κ*B-*α* degradations in the gingivomucosal tissues at eight days following ligation (Figures 2(a) and 2(b)). In addition, periodontitis caused a significant increase in the NF-*κ*B p65 levels in the nuclear fractions from of gingivomucosal tissues from operated rats (Figures 2(c) and 2(d)) compared to the gingivomucosal tissues from the contralateral side (Figures 2(c) and 2(d)). GW0742 treatment significantly prevented the periodontitis-mediated NF-*κ*B p65 expression (Figures 2(c) and 2(d)).

### 3.3. Effects of GW0742 on Cytokines Secretion, Plasma Extravasations, and Neutrophils Infiltration in Periodontitis

**Figure 3:**
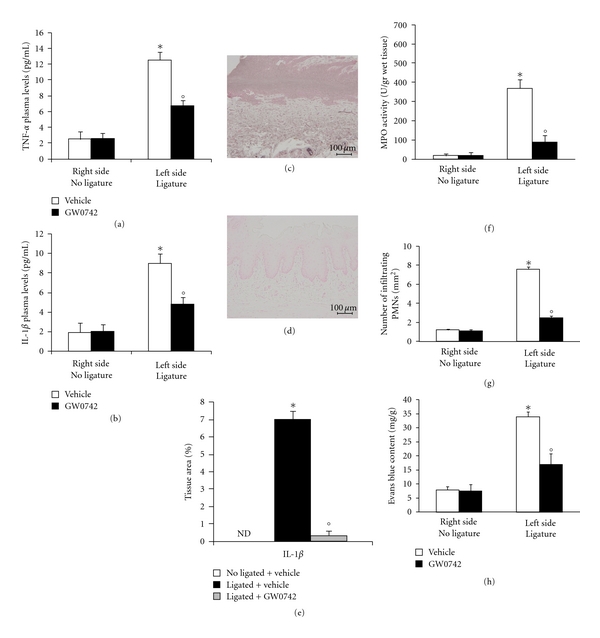
Effect of GW0742 on cytokines expression. At eight days after ligation, there was a substantial increase in TNF-*α* (a) and IL-1*β* (b) formation when compared with gingivomucosal tissue sections taken from the contralateral side (a, b). In contrast, GW0742 treatment significantly reduced TNF-*α* (a) and IL-1*β* (b) secretion. Immunohistochemical analysis of gingivomucosal tissues from ligated rats revealed positive staining for IL-1*β* (c). In gingivomucosal tissue of GW0742-treated rats no positive staining was observed for IL-1*β* (d). Densitometry analysis of immunocytochemistry photographs (*n* = 5 photos from each sample collected from all mice in each experimental group) for IL-1*β* was assessed (e). Myeloperoxidase activity (f), the total number of infiltrating leukocytes (g) and the Evans blue content (h) in gingivomucosal tissue were significantly increased by ligature compared to the contralateral side. GW0742 significantly reduced Myeloperoxidase activity (f), the total number of infiltrating leukocytes (g) and the Evans blue content (h). Data are means of mean ± S.E.M. from *N* = 10 rats for each group. *P < 0.01 versus nonligated. °*P* < 0.01 versus ligated.

To test whether GW0742 modulates the inflammatory process through the regulation of secretion of proinflammatory cytokines, we analyzed the gingivomucosal levels of the proinflammatory cytokines TNF-*α* and IL-1*β*. A substantial increase in TNF-*α* and IL-1*β* formation was observed in gingivomucosal tissues at eight days following ligation, when compared with the gingivomucosal tissues from the contralateral side (Figures 3(a) and 3(b), resp.). In contrast, a significant inhibition of these cytokines was detected in GW0742-administered animals. (Figures 3(a) and 3(b), resp.). 

As regards we analyzed, by immunohistochemical analysis, levels of IL-1*β*. Immunohistochemical analysis of gingivomucosal tissues from the contralateral side obtained from vehicle-treated rats did not reveal any immunoreactivity for IL-1*β* (data not shown). In contrast, 8 days following ligation, positive staining for IL-1*β* was found in the gingivomucosal tissues from ligature operated rats (Figure 3(c) see densitometry analysis Figure 3(e)). GW0742 treatment significantly reduced the degree of positive staining for this proinflammatory cytokine IL-1*β* (Figure 3(d) see densitometry analysis Figure 3(e)). Myeloperoxidase activity was significantly elevated at eight days after the ligature (Figure 3(f)), and GW0742 treatment significantly reduced these levels (Figure 3(f)). No significant changes of myeloperoxidase activity were observed in the gingivomucosal tissues from the contralateral side (Figure 3(f)). Quantification of infiltrating polymorphonuclear cell into gingivomucosal tissue showed that there were only a minimal number of polymorphonuclear cells in tissue from the contralateral side (Figure 3(g)). However, a large number of infiltrating polymorphonuclear cell were observed in the gingivomucosal tissue of ligated rats (Figure 3(g)). GW0742 administration significantly reduced the numbers of polymorphonuclear cell infiltrating into gingivomucosal tissue (Figure 3(g)).

Moreover, before the measurement of the Evans blue extravasations, mean arterial pressure of vehicle-treated and GW0742-treated animals was recorded. In agreement with previous studies [12], GW0742 treatment did not affect mean arterial blood pressure (vehicle treated: 128 + 6 mm Hg; *N* = 10 and GW0742 treated: 125 + 7 mm Hg; *N* = 10). Ligation significantly increased the Evans blue extravasations in gingivomucosal tissue compared to the contralateral side (Figure 3(h)). GW0742 treatment prevented this increase in the Evans blue extravasations but did not change the Evans blue content of the contralateral side (Figure 3(h)).

### 3.4. Effects of GW0742 on iNOS Expression and Nitrotyrosine Formation in Periodontitis

**Figure 4:**
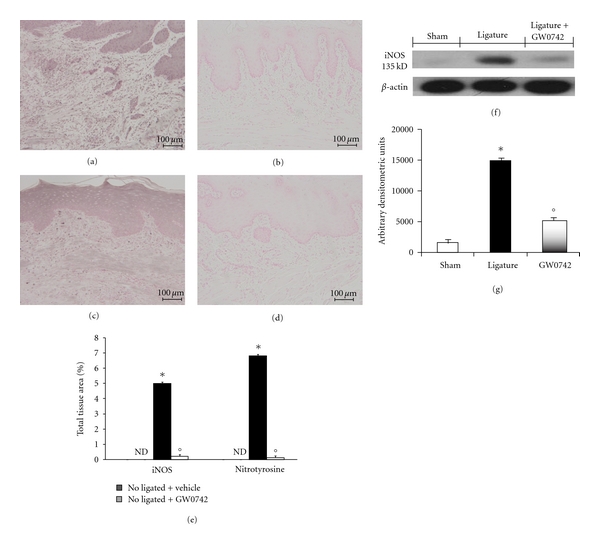
Effect of GW0742 on iNOS expression. Positive staining for iNOS (a) and nitrotyrosine (c) was observed in gingivomucosal tissue after ligature. In gingivomucosal tissue of GW0742-treated rats, no positive staining was observed for iNOS (b) and nitrotyrosine (d). Densitometry analysis of immunocytochemistry photographs (*n* = 5 photos from each sample collected from all mice in each experimental group) for iNOS and nitrotyrosine was assessed (e). Moreover a significant increase in iNOS expression, assayed by western blot analysis, was detected in the tissue from ligature-treated rats (f, g). The treatment with GW0742 significantly reduced iNOS expression (f, g). This figure is representative of at least 3 experiments performed on different experimental days. A representative blot of lysates obtained from 5 animals per group is shown, and densitometry analysis of all animals is reported. The results in (g) are expressed as mean ± S.E.M. from *n* = 5/6 gingivomucosal tissues for each group. *P < 0.01 versus nonligated group. °*P* < 0.01 versus ligated group.

Sections of gingivomucosal tissue from the contralateral side did not reveal any immunoreactivity for iNOS and nitrotyrosine, within the normal architecture (data not shown). At 8 days following ligation, positive staining for iNOS (Figure 4(a), see densitometry analysis Figure 4(e)) and nitrotyrosine (Figure 4(c), see densitometry analysis Figure 4(e)), was found in the gingivomucosal tissues from ligature-operated rats. GW0742 treatment abolished the staining for iNOS and nitrotyrosine (Figures 4(b) and 4(d); resp., see densitometry analysis Figure 4(e)). Moreover, levels of iNOS in gingivomucosal tissues were also evaluated by western blot analysis. iNOS levels were substantially increased in the gingivomucosal tissues of saline-treated rats (Figures 4(f) and 4(g)). In contrast, GW0742 treatment prevented the periodontitis-mediated iNOS expression (Figures 4(f) and 4(g)).

### 3.5. Effects of GW0742 on Bax and Bcl-2 Expression

To test whether PPAR-*β*/*δ* gene plays a role on apoptosis in gingivomucosal tissues after ligature placement, we measured Fas-L, Bax, and Bcl-2 expression by immunohistochemical analysis at eight days following ligation. 

**Figure 5:**
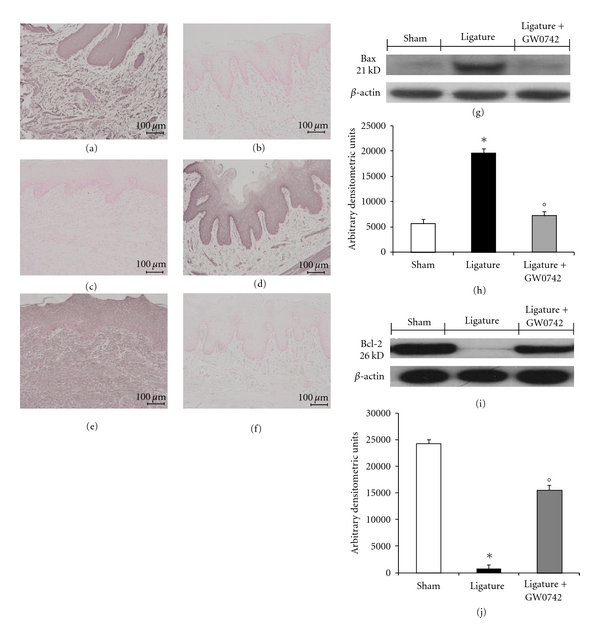
Effects of GW0742 on apoptosis. Immunohistochemistry for Bax and Fas-L showed positive staining in the gingivomucosal sections after ligature (a, e). The degree of positive staining for Bax and Fas-L was markedly reduced in GW0742-treated rats (b, f). Moreover, the degree of positive staining for Bcl-2 was markedly reduced in gingivomucosal sections obtained after ligature (c). The reduction of Bcl-2 expression was significantly attenuated by GW0742 treatment (d). In western blot analysis, basal level of Bax was present in the tissue from sham-operated rats (g, h). Bax band is more evident in the tissue from ligature-treated rats (g, h). The Bax band disappeared in the tissue from GW0742 treated (g, h). Moreover, a basal level of Bcl-2 was present in the tissue from sham-operated rats (i, j). The Bcl-2 band disappeared in the tissue from rats subjected to ligation (i, j). The Bcl-2 band is more evident in the tissue from ligated rats that received GW0742 (i, j). This figure is representative of at least 3 experiments performed on different experimental days. A representative blot of lysates obtained from 5 animals per group is shown, and densitometry analysis of all animals is reported. The results in (h, j) are expressed as mean ± S.E.M. from *n* = 5/6 gingivomucosal tissues for each group. *P < 0.01 versus nonligated group. °*P* < 0.01 versus ligated group.

No positive staining for Bax and Fas-L was observed in gingivomucosal tissues from the contralateral side obtained from vehicle-treated rats (data not shown). Immunohistochemistry for Fas-L and Bax showed positive staining in the gingivomucosal sections after ligature (Figure 5(a) and 5(e), resp.). The degree of positive staining for Fas-L and Bax were markedly reduced in GW0742-treated rats (Figures 5(b) and 5(f), resp.). To detect Bcl-2 expression, whole sample from gingivomucosal tissues of rats were also analyzed by immunohistochemical analysis. In immunohistochemical assay, the degree of positive staining for Bcl-2 was markedly reduced in gingivomucosal sections obtained after ligature (Figure 5(c)). The reduction of Bcl-2 expression caused by ligature was significantly attenuated in gingivomucosal sections from GW0742-treated rats (Figure 5(d)). A normal basal staining for Bcl-2 was observed in gingivomucosal tissues from the contralateral side obtained from vehicle-treated rats (data not shown).

Moreover, the appearance of Bax and Bcl-2 in homogenates of gingivomucosal tissues was investigated by Western blot analysis. A basal level of Bax was detectable in the homogenized gingivomucosal tissues from sham-operated animals (Figures 5(g) and 5(h)). Bax levels were substantially increased in the gingivomucosal tissues of saline-treated rats (Figures 5(g) and 5(h)). In contrast, GW0742 treatment prevented the periodontitis-mediated Bax expression (Figures 5(g) and 5(h)). A low basal level of Bcl-2 expression was detected in gingivomucosal homogenates from tissue- of sham-operated rats (Figures 5(i) and 5(j)). The expression of Bcl-2 was significantly diminished in whole extracts obtained from gingivomucosal tissues of vehicle-treated rats after ligature (Figures 5(i) and 5(j)). Treatment of rats with GW0742 significantly reduced the ligature-induced inhibition of Bcl-2 expression.

### 3.6. Effects of GW0742 Dose Response on Inflammatory Parameters

**Figure 6:**
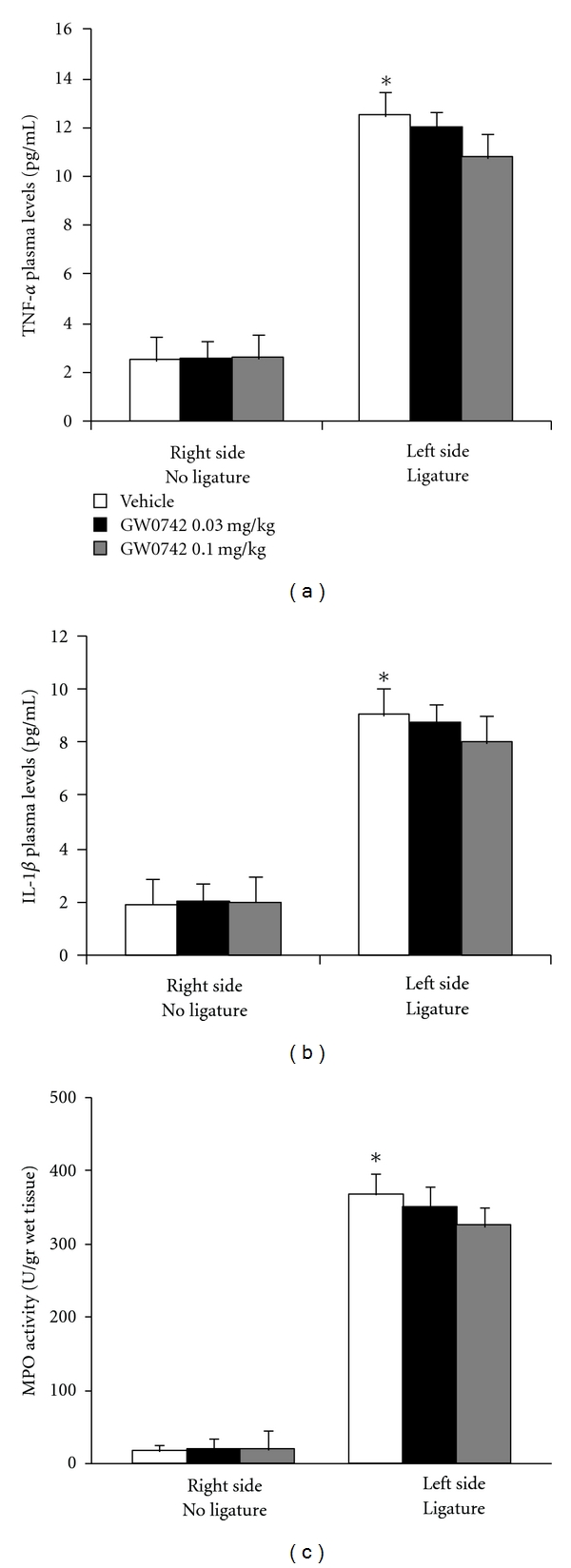
Effects of GW0742 dose response on inflammatory parameters. A substantial increase in TNF-*α*, IL-1*β* formation and MPO activity was observed in gingivomucosal tissues at eight days following ligation, when compared with the gingivomucosal tissues from the contralateral side (Figures 6(a), 6(b), and 6(c), resp.). Administration of GW0742 at different doses (0,03 and 0,1 mg/kg) did not reduce TNF-*α*, IL-1*β* formation and MPO activity in gingivomucosal tissues at eight days following ligation (Figures 6(a), 6(b), and 6(c), resp.).

To test whether different doses of GW0742 modulate the inflammatory process, we also analyzed the gingivomucosal levels of proinflammatory cytokine and neutrophil infiltration at eight days following ligation. A substantial increase in TNF-*α*, IL-1*β* formation and MPO activity was observed in gingivomucosal tissues at eight days following ligation, when compared with the gingivomucosal tissues from the contralateral side (Figures 6(a), 6(b), and 6(c), resp.). Administration of GW0742 at different doses (0,03 and 0,1 mg/kg) did not reduced TNF-*α*, IL-1*β* formation and MPO activity in gingivomucosal tissues at eight days following ligation (Figures 6(a), 6(b), and 6(c), resp.).

## 4. Discussion

In this study, we focused our attention on a potential anti-inflammatory activity role of PPAR *β*/*δ* agonist for treatment of periodontal disease. We demonstrate that a PPAR *β*/*δ* agonist, GW0742, exerts beneficial effects in a rat model of periodontitis attenuating (1) NF-*κ*B expression, (2) proinflammatory cytokines production, (3) iNOS and nitrotyrosine expression, (4) apoptosis, and (5) the degree of gingivomucosal tissues in rats subjected to ligature-induced periodontitis. All of these findings support the view that PPAR *β*/*δ* has a detrimental role in the attenuation of injury associated with periodontitis in rats. It has been known that PPAR *β*/*δ* agonists have anti-inflammatory characteristics [13]. 

Most of the anti-inflammatory effects of PPARs can probably be explained in this way. It has been known today that activation of PPARs (by either endogenous or exogenous ligands) inhibits the activation of the transcription factors including nuclear factor *κ*B (NF-*κ*B), activator protein 1 (AP-1), signal transducers and activators of transcription (STATs), and the nuclear factor of activated T cells (NFAT) [14]. This subsequently attenuates the formation of cytokines, chemokines, and adhesion molecules and, therefore, reduces excessive inflammation and tissue injury.

In this work, we confirm that PPAR-*β*/*δ* increases IkB-*α* expression, preventing nuclear p50/p65 NF-*κ*B translocation and arresting their nuclear transcriptional activity including nitric oxide synthase expression (iNOS), TNF-*α*, and IL-1*β* to name but a few. 

During the initiation and progression of periodontal disease, inflammatory cytokines are considered to play important roles. Several reports have suggested a relationship between the progression of periodontitis and the expression of interleukin-1 (IL-1), IL-6, IL-8, and tumor necrosis factor-*α* in gingival tissues [15]. There is good evidence that IL-1*β* helps to propagate the extension of a local or systemic inflammatory process [16]. Interestingly, the levels of this proinflammatory cytokine were significantly lower in the ligated rats that were treated with GW0742.

Our study also confirmed earlier findings, that one of the characteristic signs of inflammation, the Evans blue extravasation, was higher on the ligated side on the eighth day than on the opposite side. In addition, we also report in the present study that ligature-induced periodontitis in the rat results in a significant infiltration of inflammatory cells in the gingivomucosal tissues, and we also demonstrated that treatment with GW0742 reduces this inflammatory cells infiltration as assessed by myeloperoxidase. 

Neutrophils and macrophages are critical in host defense against bacterial infections. When phagocytic cell number of function is compromised, disease progression and severity are markedly increased. Periodontal disease is a common sequelae associated with altered phagocytic response. 

Neutrophils are important in periodontal disease because they control the periodontal microecology prior to involvement of chronic inflammatory cells. In contrast, monocytes and lymphocytes dictate tissue responses in periodontal microecology. It may be proposed simplistically that either hypofunction or altered PMN function or hyperfunction of monocytes/lymphocytes may result in increased susceptibility to periodontal disease. Also, though they are essential for host defense, these phagocytic cells can cause some damage to healthy tissues' Bystander effect. The junctional epithelium is particularly at risk of such damage because PMNs secrete their enzymes and toxins on bacteria which adhere to it, damaging epithelial cell underneath.

Furthermore, we found that enhanced formation of NO by iNOS may contribute to the inflammatory process. Several studies also support the conclusion that NO from iNOS has detrimental effects such as a cytotoxic action toward the host tissues, alveolar bone resorption due to the stimulating effect of nitric oxide on the activity of the osteoclasts [9, 17]. In this study, we determined the expression and, thus, the formation of iNOS, through the technique of immunohistochemistry; our results demonstrate that GW0742 treatment attenuates the expression of iNOS in periodontal tissue. Thus, the reduction of the expression of iNOS, by PPAR *β*/*δ* agonist, may contribute to the attenuation by this agent of the formation of nitrotyrosine in the periodontal tissues from ligature-treated rats. Increased nitrotyrosine staining is an indicator of “increased nitrosative stress.” 

Apoptosis, or programmed cell death, is a form of physiological cell death [18]. It is increased or decreased in the presence of infection, inflammation, or tissue remodeling. Previous studies have suggested that apoptosis is involved in the pathogenesis of inflammatory periodontal disease [19]. As apoptosis is an exceedingly complex process involving a large variety of signaling molecules; we have focused our attention on a few selective major players. From the results, we identified proapoptotic transcriptional changes, including upregulation of proapoptotic Bax and downregulation of antiapoptotic Bcl-2, using a western blot and immunohistochemistry assay. This is the first study to show that treatment with GW0742 in periodontitis inhibits and prevents the loss of the antiapoptotic pathway and, also, reduces the activation of the proapoptotic pathway by an, as yet, unidentified mechanism.

## 5. Conclusion

In conclusion, this study provides the first evidence that GW0742 causes a substantial reduction of ligature-induced periodontitis in the rat. The mechanisms underlying the protective properties of GW0742 involve modulation of transcription factors and consequent altered gene expression, resulting in downregulation of inflammation. These findings provide support that PPAR *β*/*δ* agonist, GW0742, may provide a promising approach for the treatment of periodontitis, reducing plasma extravasation and the degree of bone resorption during periodontitis.
